# Study on the Interface Constitutive Relation between Carbon Fiber Fabric and Steel

**DOI:** 10.3390/ma13153263

**Published:** 2020-07-23

**Authors:** Jianjun Shi, Bin Jia, Yinyin Ren, Xiaomei Zhang, Jian Luo

**Affiliations:** 1 Shock and Vibration of Engineering Materials and Structures Key Laboratory of Sichuan Province, Southwest University of Science and Technology, Mianyang 621010, China; stevenarmy@163.com; 2 Key Laboratory of Icing and Anti/De-icing, China Aerodynamics Research and Development Center, Mianyang Sichuan 621000, China; 3 School of Civil Engineering and Architecture, Southwest University of Science and Technology, Mianyang 621010, China; renyinyan@163.com (Y.R.); zhang010804@163.com (X.Z.); maraz5783@163.com (J.L.)

**Keywords:** carbon fiber fabric, steel, interface constitutive model, bonding strength model

## Abstract

Peeling failure at the interface is one of the main failure modes for CFRP (Carbon Fiber-Reinforced Polymer)-reinforced steel structures. However, there are very few reported studies on the bond-slip relationship at the CFRP-steel interface. A series of simple shear tests were carried out in the present paper. The influence of the fiber fabric’s width and thickness, the surface roughness of the steel sheet, and the thickness of the adhesive layer on the bonding performance of the CFRP fabric to steel interface was considered. The interface constitutive model and bonding strength model were further established under multiple factors. The results show that with the decrease of the surface roughness, the interface’ ultimate peeling load increases gradually, and the failure has a tendency to develop from a glue-steel interface to a glue-CFRP interface. The test pieces that were subjected to sand blasting obtained the peak value of the ultimate peeling load. This indicates that sand blasting can effectively enhance the interface bonding strength. The theoretical values obtained via the interface prediction model are consistent with the experimental values. This proves that the newly developed interface prediction model can effectively predict the local bonding slip and bonding strength of the interface.

## 1. Introduction

CFRP (Carbon Fiber-Reinforced Polymer) has been widely used in the reinforcement of civil engineering [1,2]. For concrete structures, remarkable results have been achieved, for example in bending reinforcement [3], shear reinforcement [4], seismic reinforcement [5], interfacial bond peeling study [6], material inspection methods [7] and durability research [8]. In recent years, CFRP has attracted more attention in relation to the strengthening and retrofitting of steel structures Compared with traditional technology, which mainly depends on welding [9,10], the CFRP strengthening steel structure has many advantages [11,12], such as a low density (about 1/4 of the steel density), high tensile properties (1000 to 5000 MPa for ordinary grade CFRP sheets), an excellent resistance to acid and alkali corrosion, a good fatigue resistance and a good creep relaxation resistance. Using CFRP hardly increases the weight of the original structure and results in a high modulus of elasticity (a Young’s modulus of about 230 GPa for an ordinary unidirectional CFRP sheet). Therefore, CFRP is a promising new type of steel structure reinforcement technology.

Studies involving CFRP in the context of the reinforcement of steel structures mainly include: external strengthening to increase the tensile bearing capacity [13]; externally reinforcing steel beams or steel-reinforced composite beams to obtain a better bending capacity [2]; externally wrapping steel columns to increase the stable [14] or compressed capacity [15]; reinforcing the steel component to improve the fatigue performance [16]; and reinforcing steel nodes to increase the bearing capacity [15]. Using CFRP to strengthen steel structures has obvious advantages, such as being convenient for construction and maintenance. However, it leads to a new problem, as the damage often occurs at the interface of CFRP and the steel structure. Ensuring effective bonding becomes a key factor for the reinforcement process. It is necessary to study the bonding properties of the CFRP-steel interface. This subject is also a hot topic in current research [17].

Similar to using CFRP-reinforced concrete, peeling failure at the interface is one of the main failure modes for CFRP-reinforced steel structures [18]. However, there are very few reported studies concerned with this problem. Most research focuses on the bonding properties of the CFRP-concrete interface, and relative theoretical models have been proposed. However, these models cannot be directly copied to the CFRP-steel interface. The former failure usually occurs in a concrete layer near the interface, while for CFRP-steel it is impossible to damage the steel surface first, but it is maybe possible to do so in a surface such as the adhesive layer, CFRP, CFRP-adhesive interface or steel-adhesive interface [19] because the strength of steel is much greater than that of the other layers. Xia and Teng [20] found that the failure occurs in the CFRP-steel interface when the thickness of the adhesive layer is 1 to 2 mm; meanwhile, when the thickness increases to 4 to 6 mm, it will turn to a mode of CFRP interlayer peeling or a mix of CFRP interlayer peeling and adhesive layer failure. Wu [21] and Al-Mosawe [22] found that if the bonding length of CFRP changes from being less than the effective bonding length to being greater than it, the failure would also change from the mode of CFRP interlayer peeling to that of the CFRP sheet being pulled off. The literature [23,24] concludes that failure is more likely to occur in the interface of steel-glue than in that of CFRP-glue. Thus, surface treating technology on the steel layer has attracted researchers’ attention as a means of avoiding failure taking place in the steel-glue interface, for example by solution cleaning, mechanical sanding, and sand blasting, etc. [18,25,26,27]. Kim [18] and Hmidan [28] found that the surface roughness of steel beams would seriously affect the bonding performance of the interface, but this study has not been discussed in depth.

The bonding strength and bond-slip relationship are the most important indicators for learning about the interfacial bonding performance. Most research focuses on evaluating the stress and bonding strength of the adhesive layer [29]. Some bonding strength models have been proposed, which can be mainly divided into two categories: strength-based [30,31] and fracture mechanics-based [32,33,34,35] prediction models. The strength-based models believe that the failure in the adhesive layer occurs when its certain strength reaches an ultimate value. For CFRP-steel bonded joints, the criterion of maximum shear stress is commonly used in failure analysis [30,31]. However, this mode cannot explain the effective bonding length, nor can it reflect the failure mechanism of CFRP-steel bonding nodes [19]. The fracture mechanics-based models believe that the bonding strength is mainly related to the interfacial fracture energy. It has been proven to be able to predict accurately the bonding strength of FRP-concrete joints and CFRP-steel joints, and it can explain the existence of effective bonding lengths well [19,31,36,37,38].

The bond-slip relationship can be regarded as a constitutive model of interface performance [39]. It determines the interface stress state, ultimate load, CFRP strain distribution, effective bonding length and other indicators [31,40]. It is a key issue in the study of CFRP-steel interface performance. However, only a few studies have focused on this problem, and there is no high-precision bond-slip constitutive model as of now. Learning from the constitutive model of CFRP-concrete, Xia and Teng [20] first proposed a bilinear constitutive model for CFRP-steel in 2005. Since then, several studies have built an approximately bilinear bond-slip relationship. Fawzia et al. [41] found that the glue layer thickness had a significant influence on the boundary slip motion, after which a modified bilinear bond-slip model for the CFRP-steel interface was proposed. Akbar [42] and Wu [16] also found that the bond-slip relationship at the interface was a bilinear distribution, and that with different kinds of adhesives the interfacial adhesion slip curve would present different developmental trends. For linear adhesive, Fernando [23] concluded that the interface constitutive model was a bilinear model and that the curve exhibited a triangular shape. For nonlinear adhesive, Dehghani [43] found that the bond-slip curve showed a plastic phase after the first elastic phase, and a trilinear model was established. Then, the model was further improved by Alemdar [44] and Al-Shawaf [45], taking the fiber fabric layer numbers and the maintenance period of the test pieces more into consideration.

In summary, there are many factors that affect the bonding performance of the interface, such as the thickness of CFRP fabric, the surface roughness of the steel sheet, the thickness of the adhesive layer, the adhesive properties and the maintenance period of the test piece. However, as the total research is in its infancy, most of the existing interface constitutive models are discussed only for a single factor, which is certainly different from the actual working conditions. Therefore, current research is regarded as being inadequate. It does not reflect the combined effects of the multiple factors on the constitutive model. In the present paper, a series of single-shear tests are performed in Section 2. The influence of the fiber fabric’s width and thickness, the surface roughness of the steel sheet, and the thickness of the adhesive layer on the bonding performance of the CFRP-steel interface is quantitatively analyzed in Section 3. The interface constitutive model and cohesive strength model under the action of multiple factors are further established in Section 4.

## 2. Materials and Methods

A simple shear test is carried out in the present paper to study the interface mechanism of CFRP fabric and steel.

Q235 steel sheets are used in the experiment. The mechanical property of Q235 steel sheet is first performed according to GB/T 228.1-2010 “Metal material tensile test Part 1: room temperature test method” [46]. The mechanical properties of CFRP fabric are tested according to GB/T 3354–1999 “Test method for tensile properties of oriented fiber reinforced plastics” [47]. The CFRP fabric and the adhesive used in the present study are all produced by Shanghai Miaohan Building Technology Co., Ltd. The adhesive consists of two components, A and B, with a weight ratio of 2:1. All material properties are shown in Table 1.

**Figure 1:**
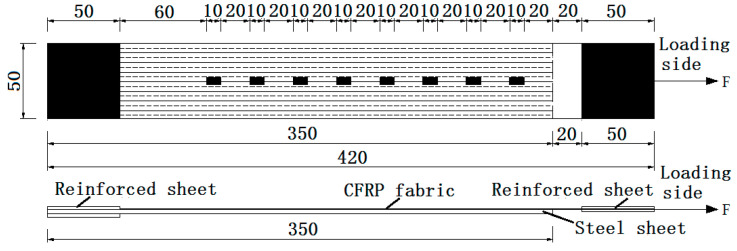
Layout of the CFRP fabric strains measuring points.

A set of eight strain gauges are attached to the CFRP fabric in each specimen. The distance between the center point of each strain gauge is 30 mm. Each strain gauge is covered with a part of silica gel to prevent contact damage. The fiber fabric is pasted along the length of the steel sheet. For better loading, a piece of steel-reinforcing sheet is pasted at both ends of the steel sheet, as shown in Figure 1.

An electro-hydraulic servo universal testing machine (Model: WAW-100, Microcomputer controlled, measuring range 10 t, made by Changchun new testing machine CO., LTD) is used as the loading device in the present experiments. Before the test, align the test piece up and down, left and right, then clamp the reinforcing sheets at both ends of the test piece onto the fixture, tightening the clamp to completely fix the test piece. The test loading method adopts displacement control with a loading rate of 0.1 mm/min. Before the experiment, the test pieces are preloaded to ensure that there is no slip between the clamp and the test piece. Then, continue to slowly load until the bonding interface is completely peeled off.

Four impact factors were considered in the test, and 48 testing specimens were prepared in total. All experiments can be divided into four groups, each group considering one of the four impact factors, including: the first group with different surface treatments for the steel sheet (sandblasted, grinding wheel-polished and no rust removal; these three surface treatment methods are defined as Class I, Class II and Class III, respectively); the second group with different CFRP fabric widths (15 mm, 30 mm, 50 mm); the third group with different CFRP fabric layers (1 layer, 2 layers, 3 layers, 4 layers) and the fourth group with different glue layer thicknesses (0.5 mm, 1.0 mm, 1.5 mm, 2.0 mm). The steel sheet used in the test has a 350-mm length, 50-mm width and 3-mm thickness, and the bonding length of the fiber fabric is 300 mm. The maintenance period of all the test pieces is seven days. The detailed information on the specimens for each testing case is shown in Table 2.

To quantify the effect of the surface roughness on the bonding performance, the surface roughness of each steel sheet undergoing surface treatment is first measured by an optical profiling instrument SuperView W1 (Measurable roughness range covers 0.1 nm to tens of microns). A parameter Sa based on the regional morphology is introduced to evaluate the roughness of the two-dimensional topography of the object’s surface, and the measurement results are shown in Table 3. It indicates that the largest value of surface roughness belongs to the unrusted steel sheet, the second one is the steel sheet polished by the grinding wheel, while the sandblasted steel sheet represents the smallest value. For the sandblasted steel sheet, a larger sand particle size will result in a larger surface roughness of the steel sheet.

## 3. Results and Discussion

### 3.1. Phenomenon and Damage Characteristics

At the initial loading stage, the load growth is slow, and the test piece does not change significantly. When the load reaches about 50% of the ultimate value, the test pieces begin to sound slightly, and the initial peeling of the CFRP fabric at the reserved port on the load side is observed. As the load continues to increase, the peeling of the interface between the CFRP fabric and steel sheet begins to progress from bottom to top. When the load reaches the ultimate peeling value, the CFRP fabric and steel sheet are completely peeled off with a loud noise, the load drops rapidly, and the test piece is destroyed. Most of the epoxy layer is attached to the CFRP fabric, and peeling occurs at the epoxy-steel interface. The experimental groups with different layer numbers and CFRP fabric widths have a similar failure mode as the one described above.

**Figure 2:**
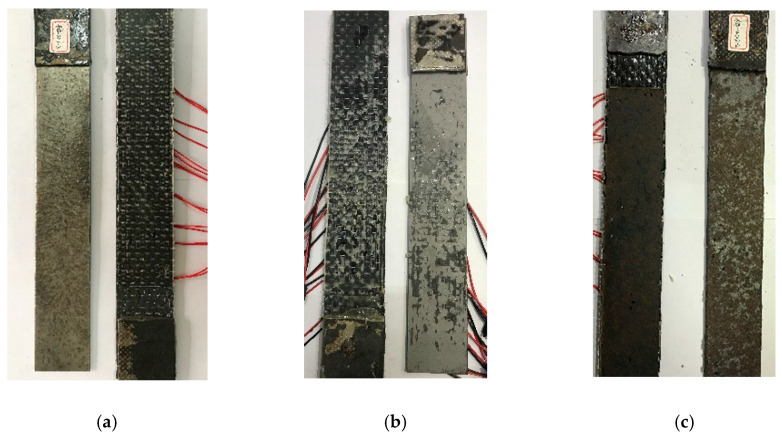
Failure mode of specimens: (**a**) Grinding wheel-polished specimen; (**b**) Sandblasted specimen; (**c**) No rust removal specimen.

For the experimental group concerned with surface treatment, the damage characteristics are slightly different, as shown in Figure 2. After the CFRP fabric of the sandblasted specimen is completely peeled off, part of the fiber and glue layer adhere to the steel plate because sandblasting increases the roughness of the steel sheet surface. Compared with the steel sheet polished by a grinding wheel, the epoxy resin has a better wettability on the sandblasted surface. After the specimen polished by the grinding wheel is damaged, the surface of the steel sheet has corrugated traces caused by the tension of the rubber layer, and no fiber tow is found. The complete peeling of the unrusted specimen occurs in the rust layer. Most of the rust adheres to the CFRP fabric and is pulled off. There is still rust on the surface of the steel sheet.

### 3.2. Characteristics of Strain Distribution in CFRP Fabric

**Figure 3:**
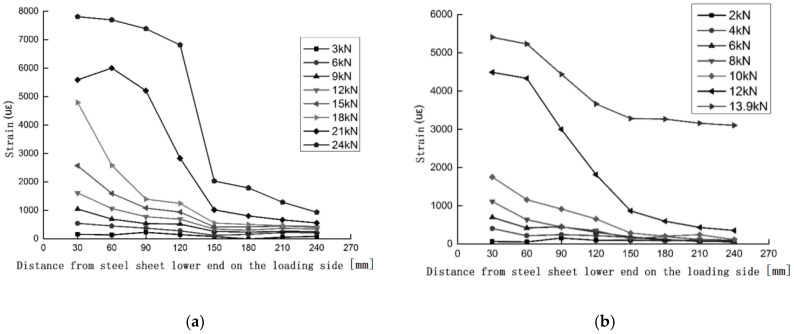
Strain-location curves of specimens (**a**) B-2-1 and (**b**) C-2-1.

The strain distribution characteristics of CFRP can reflect the transfer state of interfacial shear stress and then determine the effective bonding length of CFRP fabric and steel. As shown in Figure 3, the strain distribution curves of each specimen show that the strain value drops sharply at 150 mm from the loading end and that, when it reaches 180 mm, it is very small and basically no longer changes. In the specimens with varying number of fiber fabric layers, the effective bonding length shows a gradual increase and finally stabilizes at about 180 mm. The strain location curve also reflects the peak value of the strain of the specimen under various working conditions. It is found that after sandblasted surface treatment, the peak strain reached 7000 uε~10,000 uε, which was significantly greater than the peak strain of the other two treatment methods. Sandblasting makes the degree of infiltration of the bonding agent in the steel sheet better and gives better play to the strength of the material. When the number of fiber fabric layers increases, the peak strain value of the CFRP fabric does not increase but decreases, and the brittleness of the interface is obvious. Although the ultimate peeling bearing capacity increases with the increase of the number of fiber fabric layers, it does not increase the utilization rate of the material strength.

### 3.3. Interface Bond Slip Curve

The average shear stress at the interface between CFRP fabric and steel can be calculated by the difference method from the strain values measured at different measuring points on the CFRP fabric, as shown in Equation (1). The local slip at the interface between CFRP fabric and steel sheet is caused by the strain difference between the two materials. It can be calculated according to the strain value of each measuring point on the CFRP fabric. The slip starts on the side of the free end and gradually accumulates to the loading end [17]. When the bonding length of the CFRP fabric is long, the strain value of the steel sheet and the slip of the free end can be regarded as approximately zero. The calculation formula is shown as Equation (2).

(1) $$\tau_{i,i+1}=[E_{f}t_{f}(\varepsilon_{i+1,i}-\varepsilon_{i})]/\Delta x_{i+1,i}$$

where, $\tau_{i,i+1}$ is the average shear stress between the measuring points *i* and *i*+1; $E_{f}$ is the elastic modulus of the CFRP fabric; $t_{f}$ is the thickness of the CFRP fabric; $E_{i}$ and $\varepsilon_{i+1}$ are the strain values of the CFRP fabric at the measuring points *i* and *i*+1; and $\Delta x_{i+1,i}$ is the distance between the measuring points *i* and *i*+1.

(2) $$s_{i}=\frac{\Delta x_{i+1,i}}{2}(\varepsilon_{0}+2\sum_{j=1}^{i-1}\varepsilon_{j}+\varepsilon_{i})$$

where *S_i_* is the slip amount of the measuring point *i*; and $\varepsilon_{0}$ is the strain value of the first measuring point near the free end side.

**Figure 4:**
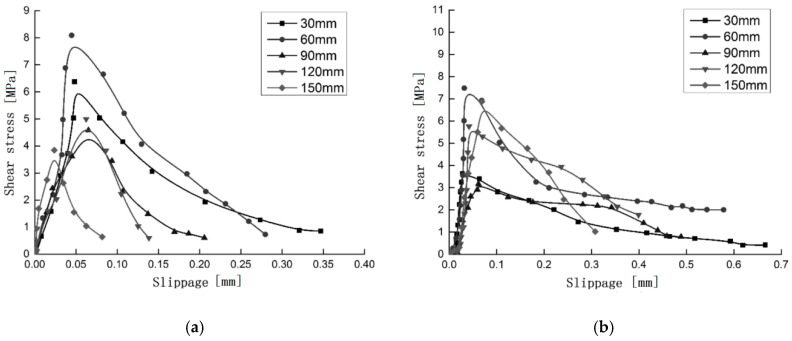
Interfacial stress-slip curves of specimens (**a**) A-3-1 and (**b**) B-1-2.

The interface bond-slip curve shown in Figure 4 is mainly composed of an ascending section and a descending section. The bond-slip curve is determined by several parameters together, in order to determine the shape and trend of the curve. These control parameters are: maximum bonding shear stress $\tau_{\max}$, peak slip *S_i_* (the slip corresponding to the maximum bonding shear stress) and maximum slip *S_max_*. The bond-slip curve can be roughly divided into the following three stages: (1) Elastic stage. The bonding force is mainly provided by the interface of the glue layer and steel sheet. The bonding shear stress increases in proportion to the slip amount. (2) The initial damage stage of the interface. At this stage, the interface bonding stiffness decreases, which is manifested by the decrease of the slope of the curve, and the interface begins to be damaged. (3) Interface peeling propagation stage. After the maximum bonding shear stress appears, the interface begins to peel off from the reserved port. The curve drops in the approximate form of an exponential function, and the amount of slip increases rapidly.

## 4. Interface Model under the Influence of Multiple Factors

### 4.1. Interface Constitutive Model

To obtain a relatively simple constitutive model, the following basic assumptions are made in the present study: (1) The tensile stress of the fiber fabric is uniform in its thickness and width; (2) The thickness of the adhesive layer is uniform in the bonding area, and only the shear stress is transmitted; (3) The specimen has no eccentric stress, and the normal stress on the cross section is evenly distributed; (4) The spacing between adjacent strain gauges is equal.

The corresponding shear stress and slip can be calculated by the strain value of each measuring point. The relationship between the strain value of the fiber fabric and the interface slip can be expressed as a function:

(3) ε=f(s)

where ε is the strain of the carbon fiber fabric, and *s* is the slippage of the interface.

Assuming that the end of the steel sheet on the side of the reserved port is taken as the starting point, the distance from each measuring point along the length of the fiber fabric to the starting point is set to be *x*; then, the first derivative of the strain value ε to the position *x* is:

(4) $$\frac{d\varepsilon }{dx}=\frac{df(s)}{ds}\cdot \frac{ds}{dx}=\frac{df(s)}{ds}\varepsilon =\frac{df(s)}{ds}f(s)$$

Substituting Equation (4) into Equation (1), we can obtain an abstract expression of the relationship between the bonding shear stress and the slip function, as shown in Equation (5). This indicates that in order to obtain the functional relationship of τ-s, the functional relationship of ε-s should first be established.

(5) $$\tau =E_{f}t_{f}\frac{d\varepsilon }{dx}=E_{f}t_{f}\frac{df(s)}{ds}f(s)$$

where $E_{f}$,$t_{f}$ are the elastic modulus and the thickness of the carbon fiber fabric.

**Figure 5:**
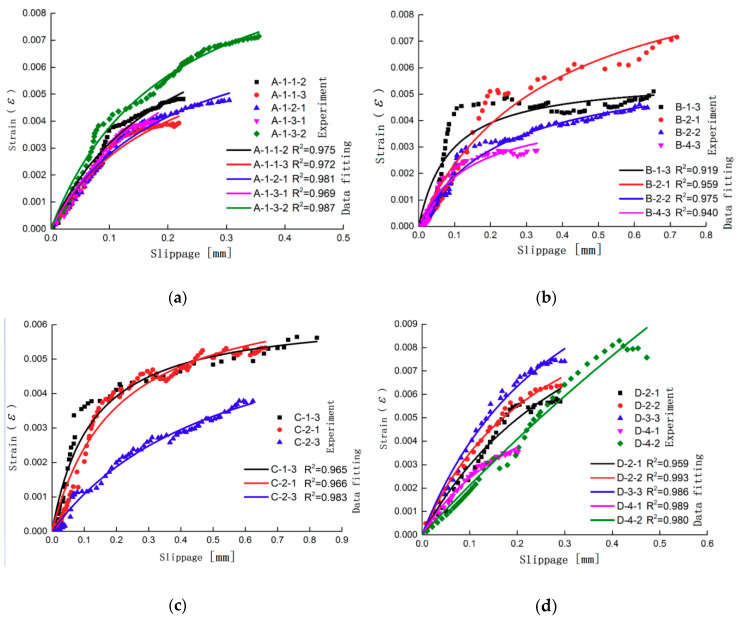
Experimental value vs. fitting curve of the strain-slip relationship: (**a**) Testing group of the sandblasted specimen; (**b**) Testing group of the specimen with different CFRP layers; (**c**) Testing group of the specimen with different CFRP fabric widths; and (**d**) Testing group of the specimen with different glue thicknesses.

Based on the strain value obtained in the test and the calculated slip value, the functional relationship between these two parameters is fitted. The strain slip test value and the fitting curve of some specimens are shown in Figure 5. This functional relationship needs to meet the following two requirements: (1) Accuracy requirement: the correlation coefficient $R^{2}$ is greater than 0.9; (2) Boundary condition requirement: the function should include the value of the (0,0) point. The final established functional relationship is shown in Equation (6).

(6) $$\varepsilon =-\frac{bs}{as+a^{2}}$$

Substituting Equation (6) into Equations (4) and (5), one obtains:

(7) $$\tau =E_{f}t_{f}\frac{d\varepsilon }{dx}=\frac{E_{f}t_{f}b^{2}s}{a{\left(a+s\right)}^{2}}$$

From the definition of the interface fracture energy, one obtains:

(8) $$G_{f}=\int_{0}^{\infty }\tau ds=\frac{E_{f}t_{f}b^{2}}{2a^{2}}$$

(9) $$b^{2}=\frac{2a^{2}G_{f}}{E_{f}t_{f}}$$

Substituting Equation (9) into Equation (7):

(10) $$\tau =\frac{2aG_{f}s}{{\left(a+s\right)}^{3}}$$

**Figure 6:**
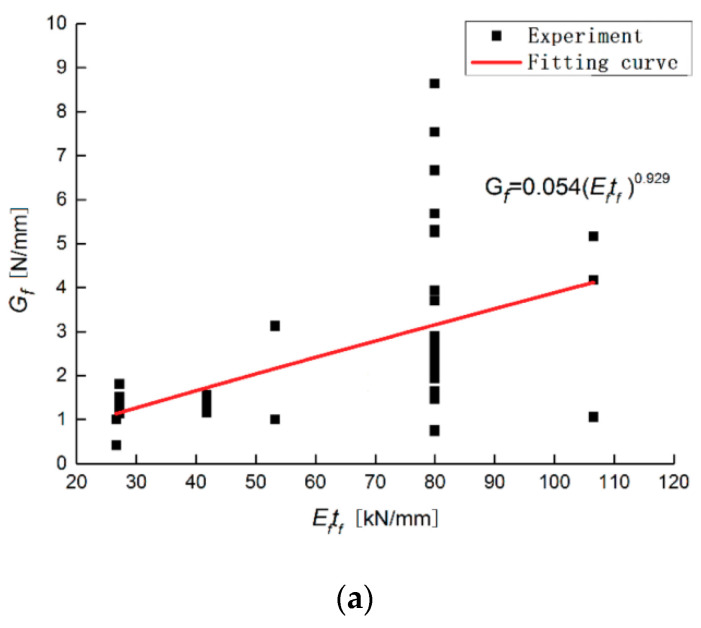

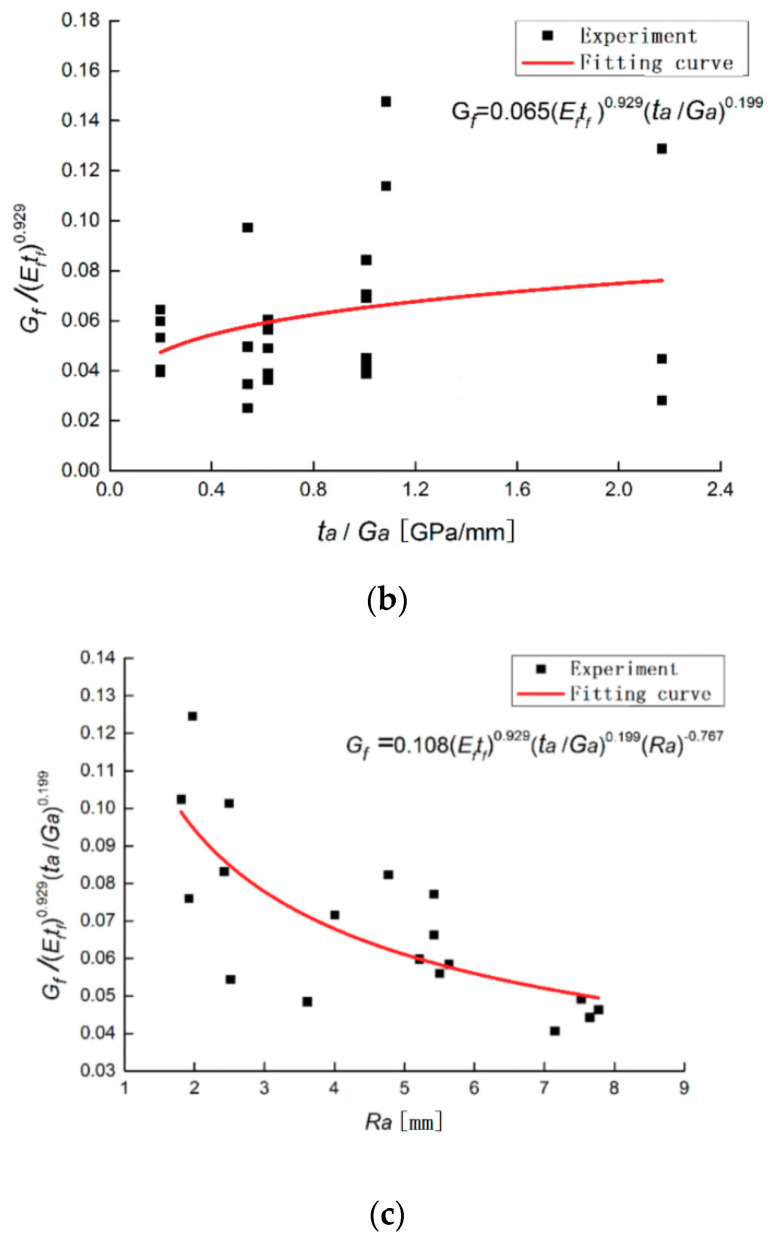
Influence of different factors on the interfacial fracture energy: (**a**) Testing group of the specimen with different CFRP fabric stiffnesses; (**b**) Testing group of the specimen with different glue thicknesses. (**c**) Testing group of the specimen with different surface roughnesses.

**Figure 7:**
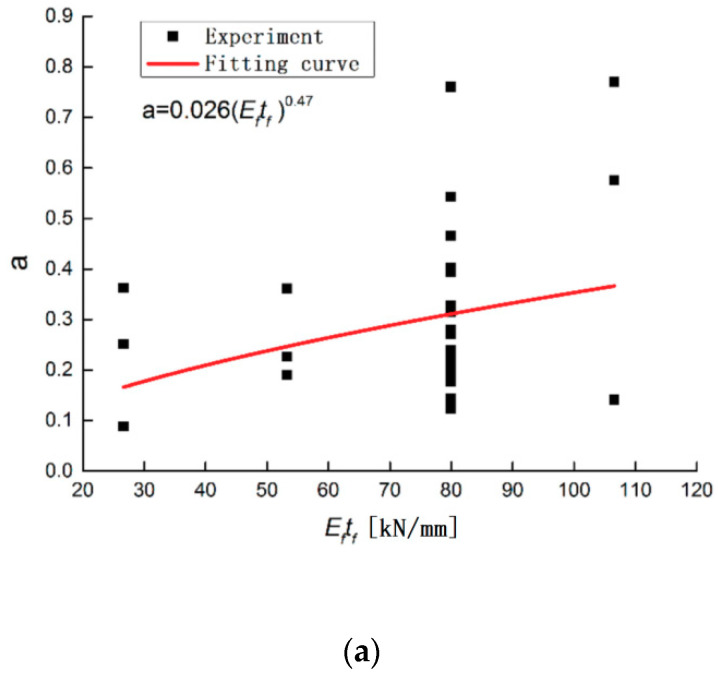

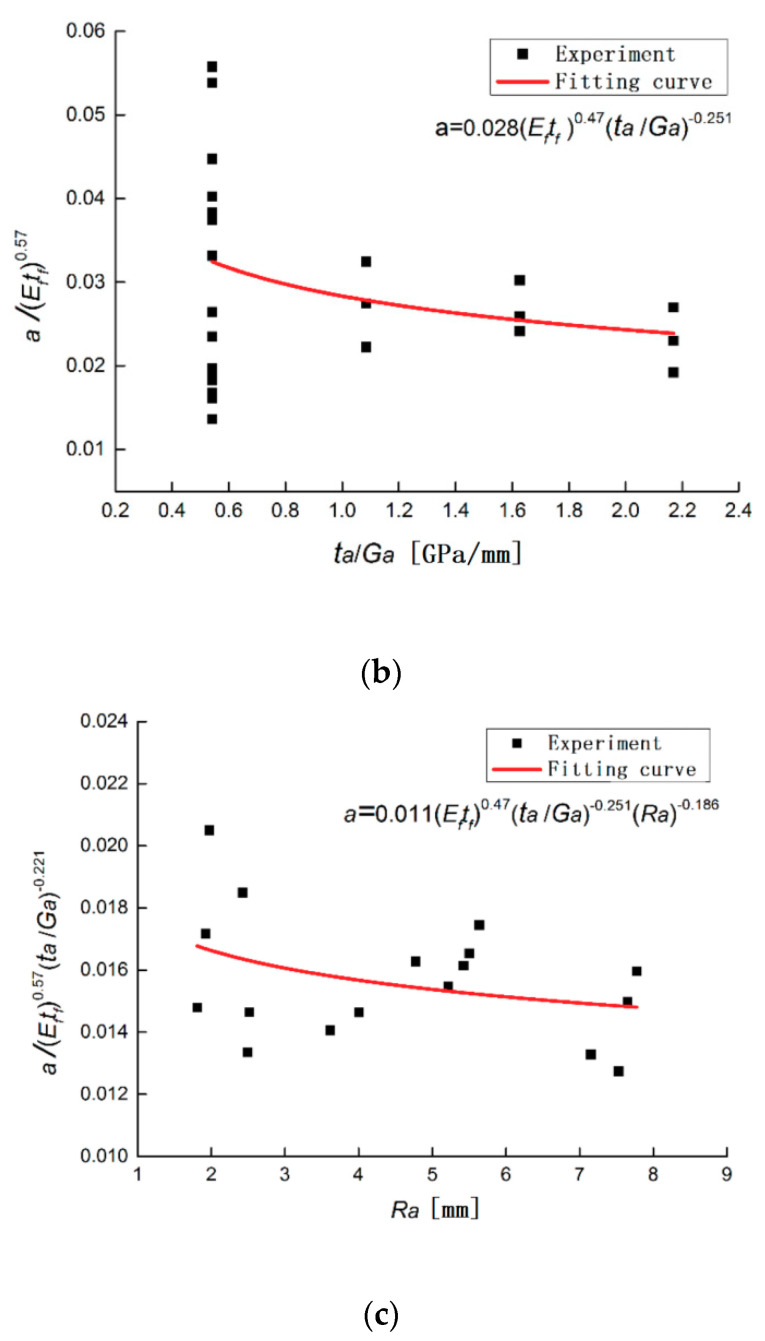
Effect of different factors on the parameter a: (**a**) Testing group of the specimen with different CFRP fabric stiffnesses; (**b**) Testing group of the specimen with different glue thicknesses. (**c**) Testing group of the specimen with different surface roughnesses.

This indicates that the functional relationship of τ-s still contains the two parameters a and $G_{f}$. To establish an interface constitutive model under the combined action of multiple factors, the relationship between these two parameters and other factors must be established first. These factors include the stiffness of the CFRP fabric, the thickness of the glue layer, and the surface roughness of the steel sheet. As shown in Figure 6 and Figure 7, the functional relationship between other factors and the two parameters a and $G_{f}$ is obtained through data fitting, as shown as Equations (11) and (12):

(11) $$G_{f}=0.118{\left(E_{f}t_{f}\right)}^{0.929}{\left(t_{a}/G_{a}\right)}^{0.199}{\left(R_{a}\right)}^{-0.767}$$

(12) $$a=0.011{\left(E_{f}t_{f}\right)}^{0.47}{\left(t_{a}/G_{a}\right)}^{-0.251}{\left(R_{a}\right)}^{-0.186}$$

where $G_{a}$ is the shear modulus of the adhesive, and $t_{a}$ is the thickness of the glue layer.

In order to obtain the maximum value of the shear stress $\tau_{\max}$ and the peak value of the slip amount, Equation (10) needs to be derived, and the result must be made equal to 0:

(13) $$s_{1}=\frac{a}{2}$$

(14) $$\tau_{\max}=\frac{8G_{f}}{27a}$$

Substituting the functional relationship between the interface fracture energy $G_{f}$, parameter a and other factors into Equation (10), the interface constitutive model under multiple factors is obtained:

(15) $$\tau =\frac{(0.0026{\left(E_{f}t_{f}\right)}^{1.40}{\left(t_{a}/G_{a}\right)}^{-0.052}{\left(R_{a}\right)}^{-0.953})s}{{\left(0.011{\left(E_{f}t_{f}\right)}^{0.47}{\left(t_{a}/G_{a}\right)}^{-0.251}{\left(R_{a}\right)}^{-0.186}+s\right)}^{3}}$$

**Figure 8:**
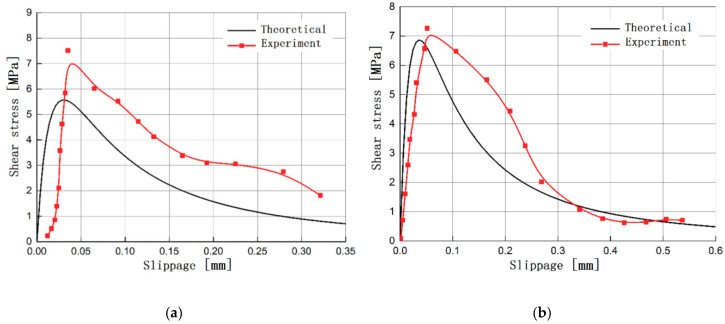
Comparison of the theoretical value calculated by the new interface constitutive model and the experimental results for specimens (**a**) B-2-1 and (**b**) C-2-3.

To verify the agreement between the theoretically derived interface constitutive model and the test, the test results are compared with the theoretical constitutive model, as shown in Figure 8. It can be seen from the figure that although there are certain differences between the predicted interface bond-slip relationship and the test curve, the overall trend of the two is in good agreement, which can be used as a reference for predicting the constitutive relationship of the interface.

Table 4 lists the theoretical prediction values and test values of all the specimens. The average of the ratio of the theoretical prediction value to the experimental value of the maximum bonding shear stress of the interface is 1.12, and the coefficient of variation is 0.177. The average value of the ratio of the theoretical prediction value to the experimental value of the peak slip is 1.09, and the coefficient of variation is 0.103. Due to certain errors caused by the experimental testing and collection, there are some cases where the ratio of the theoretical prediction values to the experimental values of several specimens is slightly larger. In general, this theoretical model can more accurately predict and characterize the interface constitutive relationship between CFRP fabric and steel.

### 4.2. Interface Bonding Strength Model

The literature [18,19] uses the energy method and the principle of force balance to derive the interface bonding strength model of FRP and concrete. The applicability of this model for FRP materials with different mechanical properties is demonstrated in the literature [20]. Similarly, this method is introduced into the calculation of the interface bonding strength between CFRP fabric and steel in the present paper. Therefore, the calculation equation for the ultimate bearing capacity of the interface $P_{u}$ is:

(16) $$P_{u}=b_{f}E_{f}t_{f}\varepsilon_{\max}=-\frac{b}{a}b_{f}E_{f}t_{f}$$

Substituting Equations (9) and (12) into the above Equation (16), the interface bonding strength model of CFRP fabric and steel is obtained as follows:

(17) $$P_{u}=0.486b_{f}\sqrt{{\left(E_{f}t_{f}\right)}^{1.929}{\left(t_{a}/G_{a}\right)}^{0.199}{\left(R_{a}\right)}^{-0.767}}$$

**Figure 9:**
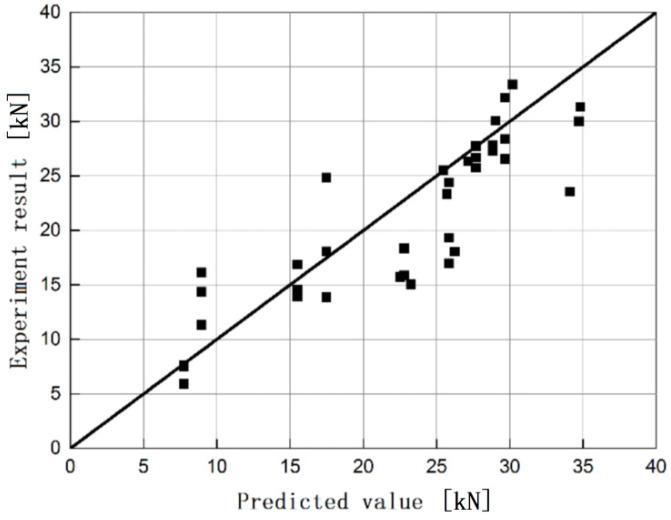
Comparison of the predicated interfacial ultimate bearing capacity and the experimental results.

It can be seen from Figure 9 that the data points are more evenly distributed on both sides of the straight line, indicating that the theoretically predicted value of the ultimate bearing capacity agrees well with the experimental value. The interface bonding strength model of CFRP fabric and steel obtained by derivation can be used to calculate the predicted ultimate bearing capacity of each test piece. When compared with the experimental values, which are listed in Table 4, the average of the ratio of the two values is 0.93, and the coefficient of variation is 0.208.

## 5. Conclusions

In order to learn about the influence of multiple factors on the interface constitutive model and the interface bonding strength model, a simple shear test of CFRP fabric and a steel sheet composite was carried out in the present paper. The failure mode, the ultimate peeling load and the relationship of bonding shear stress vs. slippage were observed. An interface model was established under the influence of multiple factors. Finally, its results were compared with the experiment. All studies led to the following conclusions:

(1) Various levels of surface roughness can be obtained by treating the steel sheet surface. The test shows that as the roughness decreases, the failure location tends to develop from the adhesive-steel interface to the adhesive-CFRP interface.
(2) The interface ultimate peeling load increases nonlinearly with the increase of the CFRP fabric width, number of layers and adhesive layer thickness. The peeling load at the interface of the sandblasted test piece obtains the maximum value. This result indicates that sandblasting treatment can effectively enhance the bonding strength of the interface.
(3) Considering the effects of multiple factors, the interface constitutive model and the interface bonding strength model are established in the present paper. A comparison with the experimental results shows that the predicted values of the two models are in good agreement with the experimental values. This leads to the conclusion that the newly built models can effectively predict the interface peeling load and the relationship between the local bonding shear stress and slippage.

## Figures and Tables

**Table 1:** Properties of the experimental materials.

Test Material	Elastic Modulus/[GPa]	Yield Strength/[MPa]	Yield Strain/uε	Ultimate Tensile Strength/[MPa]	Elongation /%	Thickness/[mm]
Q235 Steel sheet	192.97	220.92	1145	360	35	3
CFRP fabric	240	—	—	3325	1.74	0.111
Epoxy resin	2.47	—	—	50.39	1.9	—

**Table 2:** Specimens’ details in each testing case.

Test Condition			Serial Number	Specimen Number	CFRP Layer Number	CFRP Width/mm	Surface Treatment	Maintenance Period
**steel sheet surface treatment**	sandblasted	32 sieve number	A-1-1	3	3	50	Class I	7d
		60 sieve number	A-1-2	3	3	50	Class I	7d
		120 sieve number	A-1-3	3	3	50	Class I	7d
	grinding wheel-polished		A-2	3	3	50	Class Ⅱ	7d
	no rust removal		A-3	3	3	50	Class Ⅲ	7d
**CFRP fabric layer number**	1 layer		B-1	3	1	50	Class Ⅱ	7d
	2 layer		B-2	3	2	50	Class Ⅱ	7d
	3 layer		B-3	3	3	50	Class Ⅱ	7d
	4 layer		B-4	3	4	50	Class Ⅱ	7d
**CFRP fabric width**	15 mm		C-1	3	3	15	Class Ⅱ	7d
	30 mm		C-2	3	3	30	Class Ⅱ	7d
	50 mm		C-3	3	3	50	Class Ⅱ	7d
**glue layer thickness**	0.5 mm		D-1	3	3	50	Class Ⅱ	7d
	1.0 mm		D-2	3	3	50	Class Ⅱ	7d
	1.5 mm		D-3	3	3	50	Class Ⅱ	7d
	2.0 mm		D-4	3	3	50	Class Ⅱ	7d

**Table 3:** Surface roughness of the steel sheet in the first group undergoing different surface treatments.

Specimen Serial Number	Average Value of Sa for Each Measurement Point/[µm]	Specimen Serial Number	Average Value of Sa for Each Measurement Point/[µm]
A-1-1-1	4.774	A-1-3-3	1.974
A-1-1-2	4.007	A-2-1	5.637
A-1-1-3	3.617	A-2-2	5.504
A-1-2-1	2.515	A-2-3	5.215
A-1-2-2	2.494	A-3-1	7.153
A-1-2-3	2.425	A-3-2	7.777
A-1-3-1	1.924	A-3-3	7.65
A-1-3-2	1.809	—	—

**Table 4:** Comparison of the theoretical predicted value calculated by the new interface constitutive model (Subscripted, “*p*”) and the experimental results (Subscripted, “*e*”).

Specimen Number	$G_{f}$	$\tau_{\max,e}/[\mathrm{MPa}]$	$\tau_{\max,p}/[\mathrm{MPa}]$	$\frac{\tau_{\max,e}}{\tau_{\max,p}}$	$s_{1,e}/[\mathrm{mm}]$	$s_{1,p}/[\mathrm{mm}]$	$\frac{s_{1,e}}{s_{1,p}}$	$P_{u,e}/[\mathrm{kN}]$	$P_{u,p}/[\mathrm{kN}]$	$\frac{P_{u,e}}{P_{u,p}}$
A-1-1	2.078	9.08	7.95	1.14	0.043	0.039	1.10	29.93	28.79	1.04
A-1-2	3.050	8.28	10.64	0.78	0.035	0.043	0.81	30.66	34.82	0.88
A-1-3	3.739	10.17	12.41	0.82	0.056	0.045	1.10	31.35	38.65	0.81
A-2	1.672	9.57	7.75	1.23	0.044	0.037	1.08	22.29	25.85	0.86
A-3	1.300	7.6	5.58	1.36	0.042	0.035	1.08	14.7	22.79	0.65
B-1	1.667	6.53	5.08	1.29	0.032	0.022	1.28	13.39	8.96	1.49
B-2	1.302	7.45	6.6	1.13	0.035	0.030	0.97	18.92	17.48	1.08
B-3	1.672	9.57	7.75	1.23	0.044	0.037	1.08	22.29	25.85	0.86
B-4	2.184	10.38	8.7	1.19	0.051	0.042	1.14	21.11	34.11	0.62
C-1	1.672	11.31	7.75	1.46	0.044	0.037	1.11	7.02	7.54	0.93
C-2	1.672	7.7	7.75	0.99	0.044	0.037	1.08	15.11	15.51	0.97
C-3	1.672	9.57	7.75	1.23	0.044	0.037	1.08	22.29	25.85	0.86
D-1	1.672	9.57	7.75	1.23	0.044	0.037	1.08	22.29	25.85	0.86
D-2	1.919	10.72	9.22	1.16	0.038	0.031	1.12	26.72	27.70	0.96
D-3	2.018	9.48	11.07	0.86	0.035	0.028	1.14	27.53	28.84	0.95
D-4	2.203	10.49	12.6	0.83	0.042	0.026	1.36	29.04	29.67	0.98
